# Seaweed polysaccharide relieves hexavalent chromium-induced gut microbial homeostasis

**DOI:** 10.3389/fmicb.2022.1100988

**Published:** 2023-01-16

**Authors:** Jinghao Mu, Zhenhuan Guo, Xiujun Wang, Xuefei Wang, Yunxing Fu, Xianghui Li, Fuli Zhu, Guangyuan Hu, Xia Ma

**Affiliations:** ^1^Department of Urology, Chinese PLA General Hospital, Beijing, China; ^2^Department of Urology, The Seventh Medical Center of Chinese PLA General Hospital, Beijing, China; ^3^Zhengzhou Key Laboratory of Immunopharmacology of Traditional Chinese Veterinary Medicines, Henan University of Animal Husbandry and Economy, Zhengzhou, Henan, China

**Keywords:** chromium, seaweed polysaccharides, gut microbiota, bacteria, heavy metals

## Abstract

Heavy metals released in the environment pose a huge threat to soil and water quality, food safety and public health. Additionally, humans and other mammals may also be directly exposed to heavy metals or exposed to heavy metals through the food chain, which seriously threatens the health of animals and humans. Chromium, especially hexavalent chromium [Cr (VI)], as a common heavy metal, has been shown to cause serious environmental pollution as well as intestinal damage. Thus, increasing research is devoted to finding drugs to mitigate the negative health effects of hexavalent chromium exposure. Seaweed polysaccharides have been demonstrated to have many pharmacological effects, but whether it can alleviate gut microbial dysbiosis caused by hexavalent chromium exposure has not been well characterized. Here, we hypothesized that seaweed polysaccharides could alleviate hexavalent chromium exposure-induced poor health in mice. Mice in Cr and seaweed polysaccharide treatment group was compulsively receive K2Cr2O7. At the end of the experiment, all mice were euthanized, and colon contents were collected for DNA sequencing analysis. Results showed that seaweed polysaccharide administration can restore the gut microbial dysbiosis and the reduction of gut microbial diversity caused by hexavalent chromium exposure in mice. Hexavalent chromium exposure also caused significant changes in the gut microbial composition of mice, including an increase in some pathogenic bacteria and a decrease in beneficial bacteria. However, seaweed polysaccharides administration could ameliorate the composition of gut microbiota. In conclusion, this study showed that seaweed polysaccharides can restore the negative effects of hexavalent chromium exposure in mice, including gut microbial dysbiosis. Meanwhile, this research also lays the foundation for the application of seaweed polysaccharides.

## Introduction

Industrial production releases a large amount of metal pollutants every year, such as lead, chromium, and copper, which are considered to be vital factors causing environmental contamination and animal metal poisoning (Wen et al., 2019; Zhao et al., 2019; Wang F. et al., 2022). Chromium is one of the most common heavy metals, which is widely used in leather, fuel and steel production (Mamais et al., 2016; Kapoor et al., 2022). Early surveys indicated that the global annual consumption of chromium is more than 200,000 tons and its demand is still increasing (Li A. et al., 2021). However, large amounts of chromium waste may be directly discarded and reach the environment through multiple ways, seriously threatening the surrounding water and soil health (Vaiopoulou and Gikas, 2020; Prasad et al., 2021). Importantly, the released chromium could accumulate in soil, water and plants then transfer to aquatic and terrestrial animals *via* food chain, posing a serious threat to human health and food safety (Nguyen et al., 2017; Lee C. P. et al., 2019). Previous studies indicated that long-term exposure to hexavalent chromium can cause parenchymal organ injury such as gastrointestinal tract, liver, and kidney. Additionally, hexavalent chromium has also been shown to be associated with cancer, asthma and gut microbial dysbiosis (Yang Q. et al., 2020; Monteiro et al., 2018; Shaw et al., 2019).

Gut microbiota is a complicated and dynamic microecosystem that consists of approximately 100 trillion microorganisms involving over 2,000 diverse species (Egerton et al., 2018; Feng et al., 2018; Morris, 2018). The gut microbiota exhibits a symbiotic relationship with the host, exerting positive effects on host metabolism, intestinal homeostasis and immune system maturation (Yu et al., 2020; Zhang L. et al., 2021). Moreover, the other well-understood contributions of the gut microbial community is its key roles in the intestinal barrier maintenance and immune system maturation, which contribute to protecting the host from invasion by infectious pathogens (Sun et al., 2022; Wang R. et al., 2022). As essential biochemical converters, gut microbiota can also convert food into nutrients and metabolites (Zhang L. et al., 2021; Zhang X. et al., 2021; Yang J. et al., 2022). However, many factors associated with hosts and environment such as aging, oxidative stress, antibiotics and heavy metal could affect intestinal homeostasis and even induce gut microbial dysbiosis (Xia et al., 2018; Kakade et al., 2020; Ma et al., 2022). Numerous studies provided supporting evidence that gut microbial dysbiosis could impair intestinal mucosal barrier and gut mucosal immune system, potentially causing severe gastrointestinal infection, diarrhea, and colonitis (Liu et al., 2019; Li Y. et al., 2021; Xu et al., 2022). Additionally, gut microbial dysbiosis can also extend its negative effects beyond the gastrointestinal system and result in extraintestinal diseases such as autism, diabetes, obesity and NAFLD (Yang et al., 2021; Wan and Ma, 2022; Ye et al., 2022). Considering the systemic effects of gut microbial dysbiosis, it is also considered as a emerging participator in the pathophysiology of many diseases (Crusell et al., 2018).

Supplementation with antioxidants is regarded as a vital way to mitigate metal poisoning because metal contaminants can cause oxidative stress and decreased antioxidant capacity (He et al., 2020; Yang Y. et al., 2020; Paithankar et al., 2021). Currently, polysaccharides extracted from animals and plants have been shown to be promising antioxidants (Chen and Huang, 2018, 2019). Among many types of polysaccharides, seaweed polysaccharide has attracted mounting attention own to its several health benefits to the host (Tanna and Mishra, 2019; Bauer et al., 2021). Numerous studies indicated that seaweed polysaccharide has anti-inflammatory, antiviral, immunomodulatory and anti-tumor effects (Lomartire and Goncalves, 2022). Moreover, recent research on seaweed polysaccharide also showed its vital roles in the gastrointestinal disease and improve antioxidant ability (Fu et al., 2021). Although increasing evidence showed the positive role of seaweed polysaccharide on the host health, it remains unclear whether seaweed polysaccharide can alleviate gut microbial imbalance caused by hexavalent chromium. Thus, we investigated the protective effect of seaweed polysaccharide on hexavalent chromium induced gut microbial imbalance.

## Materials and methods

### Animal experiments

Sixty 28-day-old Kunming mice with similar weight and background were used for this research. These selected mice were housed in a standard environment and health assessments were performed on all mice to ensure that the experiments ran smoothly. After acclimatization for 3 days, these mice were randomly divided into three groups namely control group (Con), Cr (VI)-induced group (Cr), seaweed polysaccharide treatment group (SP, 200 mg/kg). The dosage of seaweed polysaccharide and Cr (VI) refers to the previous research with slight improvements (Ben et al., 2016; Fu et al., 2021). The proportion of male and female in each groups was 1:1. The mice in the Con, Cr and SP groups were provided adequate feed and water. In addition, mice in Cr (VI) and SP treatment group was compulsively receive K_2_Cr_2_O_7_ (75 mg/kg). Moreover, the SP treatment group was compulsively gavaged with 0.2 ml of SP. On the day 29 of the experiment, we euthanized all the mice and collected colonic contents. The collected samples were snap-frozen in liquid nitrogen and stored at-80°C until further investigation.

### DNA extraction and illumine MiSeq sequencing

The acquired samples of each group were separately homogenized and then performed DNA extraction using QIAamp DNA Mini Kit (QIAGEN, Hilden, Germany) following suggested instructions of manufacturer. After ensuring the extracted DNA met the requirements for subsequent analysis, we amplified the V3/V4 variable regions using the primers (338F: ACTCCTACGGGAGGCAGCA and 806R: GGACTACHVGGGTWTCTAAT) synthesized from conserved regions. The conditions and volumes of PCR reactions were determined as per previous studies (Zhang et al., 2020). Before building the libraries, some processing products including fragment recovery, quantitation and quality appraisal were performed to obtain qualified products. The constructed libraries were subsequently performed quality evaluation. The libraries with only one peak and concentration greater than 2 nM were considered qualified. The qualified libraries were subjected to paired-end sequenced (2 × 300 bp) on MiSeq sequencing machine following the standard protocols. The original data containing short sequences, chimera and mismatched primers preduced from amplicon sequencing were performed quality evaluation and filtration to acquire effective sequence. The effective sequences were clustered and OTUs partitioned based on 97% similarity. To further dissect the effects of thiram exposure on gut microbiota, we calculated five alpha diversity indices and generated PCoA plots that reflected beta diversity. The differential bacteria were identified through the Metastats analysis and LEfSe. *p*-values (means ± SD) <0.05 were considered statistically significant.

## Results

### Data collection and analysis

**Figure 1:**
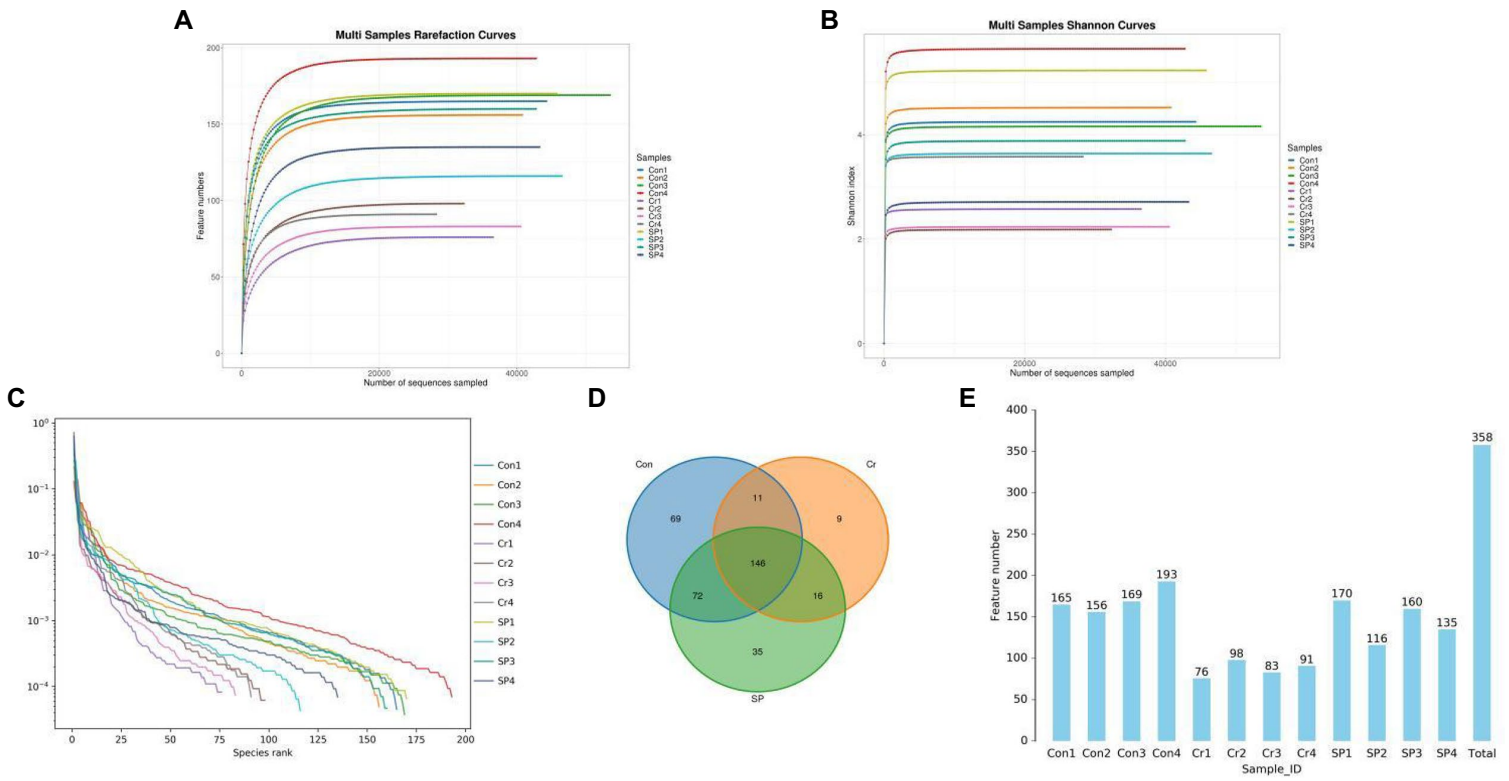
Feasibility assessment and OTU distribution. **(A,B)** Rarefaction curves. **(C)** Rank abundance curve. **(D)** Venn diagram. **(E)** OTUs distribution histogram.

To investigate the protective effect of seaweed polysaccharide on Cr (VI)-induced mice, we explored changes in the gut microbiota of mice during polysaccharide supplementation. Results indicated that a total of 735,152 (Con = 256,535, Cr = 222,963, SP = 255,654) raw sequences were obtained from three groups (Table 1). Subsequently, we performed quality assessment on the raw data and obtained 502,400 (Con = 183,752, Cr = 138,389, SP = 180,259) valid sequences. Results of the rarefaction curves, which can reflect the sequencing depth, show that the species coverage and sequencing depth are qualified (Figures 1A–C). The valid sequences of three groups were clustered into 358 OTUs (Con = 298, Cr = 182, SP = 269), ranging from 76 to 193 OTUs per sample (Figures 1D,E). Furthermore, the Con, Cr and SP groups have 69, 9, and 35 unique OTUs, respectively.

**Table 1:** The raw sequence information generated from amplicon sequencing.

Sample	Raw reads	Clean reads	Denoised reads	Merged reads	Effective reads	Effective (%)
Con1	63,877	48,706	47,825	46,605	44,712	69.99
Con2	52,984	42,083	41,786	41,500	41,298	77.94
Con3	68,338	56,977	56,209	55,487	54,201	79.31
Con4	71,336	54,244	51,810	48,256	43,541	61.03
Cr1	57,184	45,048	44,317	43,248	36,645	64.08
Cr2	43,218	33,053	32,877	32,630	32,505	75.21
Cr3	64,568	50,981	50,095	48,996	40,654	62.96
Cr4	57,993	44,484	43,224	41,448	28,585	49.29
SP1	72,200	55,611	54,193	52,194	46,092	63.83
SP2	59,875	48,195	47,982	47,741	46,954	78.42
SP3	68,315	52,716	50,800	48,336	43,504	63.68
SP4	55,264	44,248	44,016	43,778	43,709	79.09

### Seaweed polysaccharide recovered the changes of gut microbial diversity induced by Cr (VI)

**Figure 2:**
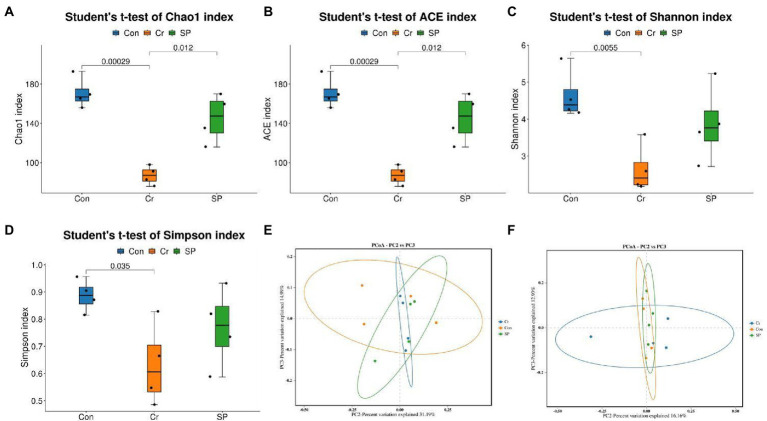
Seaweed polysaccharide administration restored the changes in gut microbial diversity induced by hexavalent chromium exposure. **(A)** Chao1. **(B)** ACE. **(C)** Shannon. **(D)** Simpson. **(E,F)** PCoA plots based on the weighted and unweighted UniFrac distance.

We further calculated changes in gut microbial diversity based on the abundance of OTUs in each sample. Results of Good’s coverage indicated that almost all bacterial phenotypes were found in this amplicon sequencing. The gut microbial diversity indices such as Chao1 (170.75 ± 15.79 vs. 87.00 ± 9.55, *p* = 0.00029), ACE (170.75 ± 15.79 vs. 87.00 ± 9.55, *p* = 0.00029), Shannon (4.64 ± 0.68 vs. 2.64 ± 0.64, *p* = 0.0055), and Simpson (0.88 ± 0.060 vs. 0.63 ± 0.15, *p* = 0.035) in the hexavalent chromium exposure group were significantly lower than those in the control group, indicating that hexavalent chromium markedly reduced the diversity and abundance of gut microbiota. However, seaweed polysaccharide administration reversed the hexavalent chromium-induced decrease in gut diversity indices (Figures 2A–D). PCoA plots indicated that all the samples were clustered together, indicating no differences in the major components of the gut microbiota (Figures 2E,F).

### Seaweed polysaccharide altered gut microbial composition in Cr (VI)-induced mice

**Figure 3:**
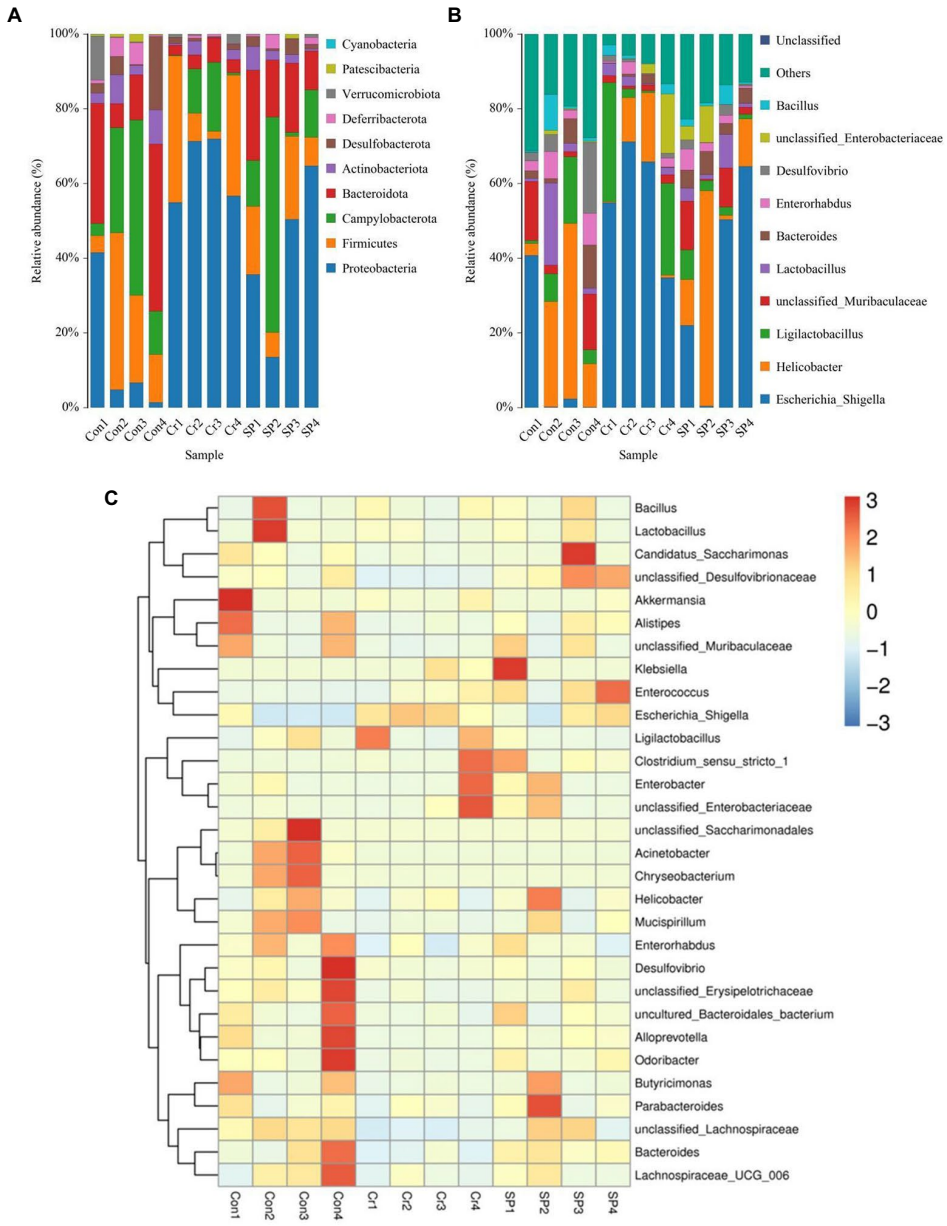
Seaweed polysaccharide administration restored gut microbial composition in hexavalent chromium-induced mice. **(A,B)** The preponderant bacteria at the phylum and genus levels. **(C)** Heat map of bacterial distribution.

The gut microbial composition and abundance in different taxonomical levels were evaluated and observed significant variations. In this amplicon sequencing, a total of 10 phyla and 91 genera were identified in 12 samples, ranging from 7 to 10 phyla and 37 and 69 genus per sample. *Proteobacteria* (13.49, 40.43%), *Campylobacterota* (23.70, 21.54%), *Firmicutes* (20.47, 13.58%) and *Bacteroidota* (23.43, 17.17%) were the most preponderant bacteria in Con and SP groups, whereas *Proteobacteria* (64.11%), *Campylobacterota* (8.41%), *Firmicutes* (19.43%), and *Actinobacteriota* (1.58%) were abundantly present in the Cr groups (Figure 3A). However, the abundances of *Deferribacterota* (3.18, 0.42, 1.57%), *Verrucomicrobiota* (2.94, 0.82, 0.22%), *Patescibacteria* (0.97, 0.021, 0.31%), and *Cyanobacteria* (0.033, 0.00, 0.00%) are lower in Con, Cr and SP groups. *Escherichia_Shigella* (10.71, 33.45%) and *Helicobacter* (23.70, 21.54%) were the most dominant genus in the Con and SP groups, whereas *Escherichia_Shigella* (57.75%) and *Ligilactobacillus* (14.24%) were abundantly present in the Cr group (Figure 3B). However, the proportions of *Enterorhabdus* (4.94, 1.41, 2.71%), *Desulfovibrio* (6.07, 0.89, 1.43%), *unclassified_Enterobacteriaceae* (0.43, 4.05, 3.52%), and *Bacillus* (2.63, 1.57, 2.06%) were lower in gut microbiota of Con, Cr, and SP groups. The specific bacterial species and abundance are also shown in the heatmap (Figure 3C).

**Figure 4:**
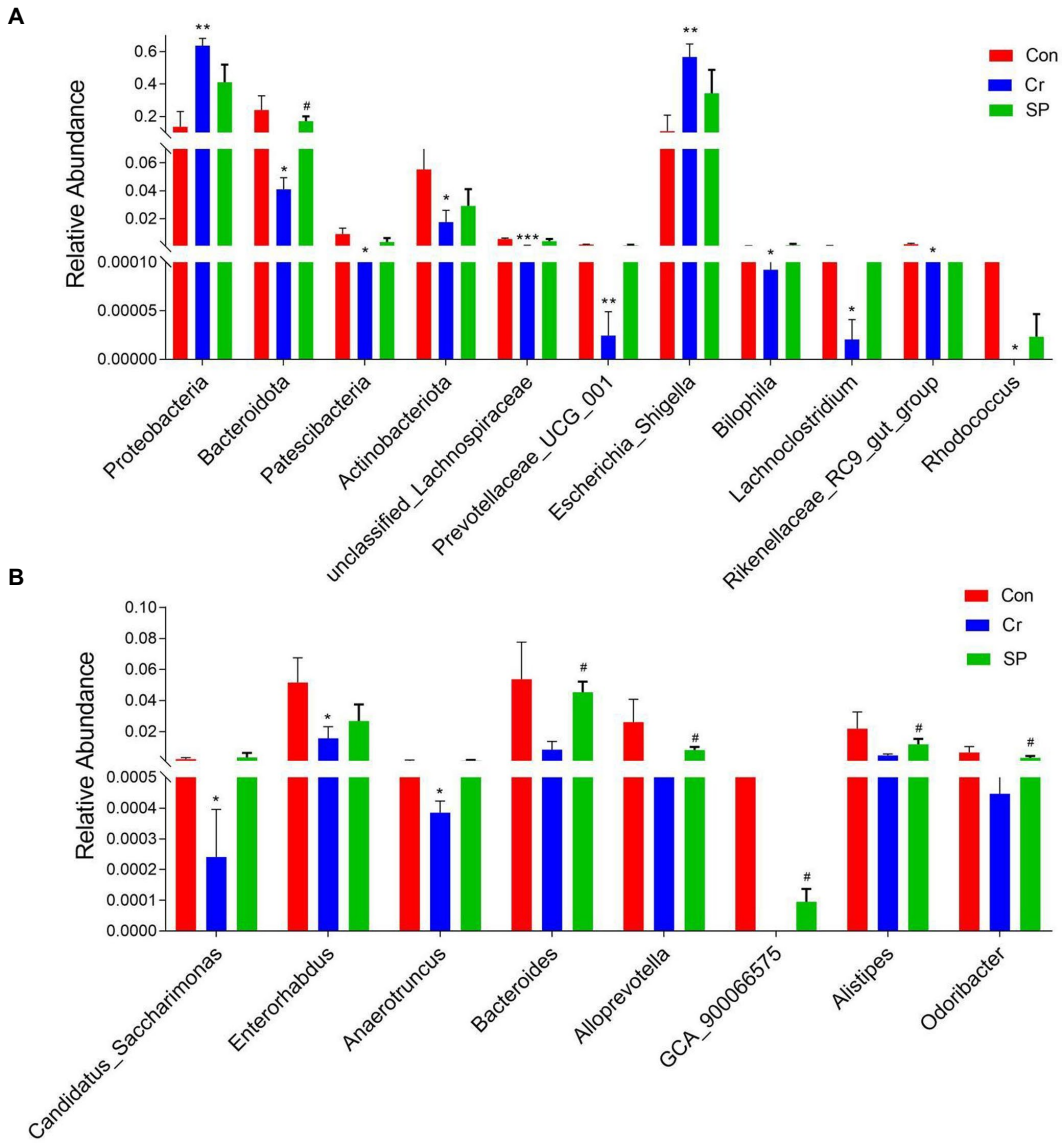
Statistical analysis of differential bacteria. **(A)** Relative Abundance at genus level. **(B)** Relative Abundance at phylum level. All data was indicated as mean ± SD. *p < 0.05, **p < 0.01, ***p < 0.001 compared with Con group, ^#^*p* < 0.05 compared with Cr group.

**Figure 5:**
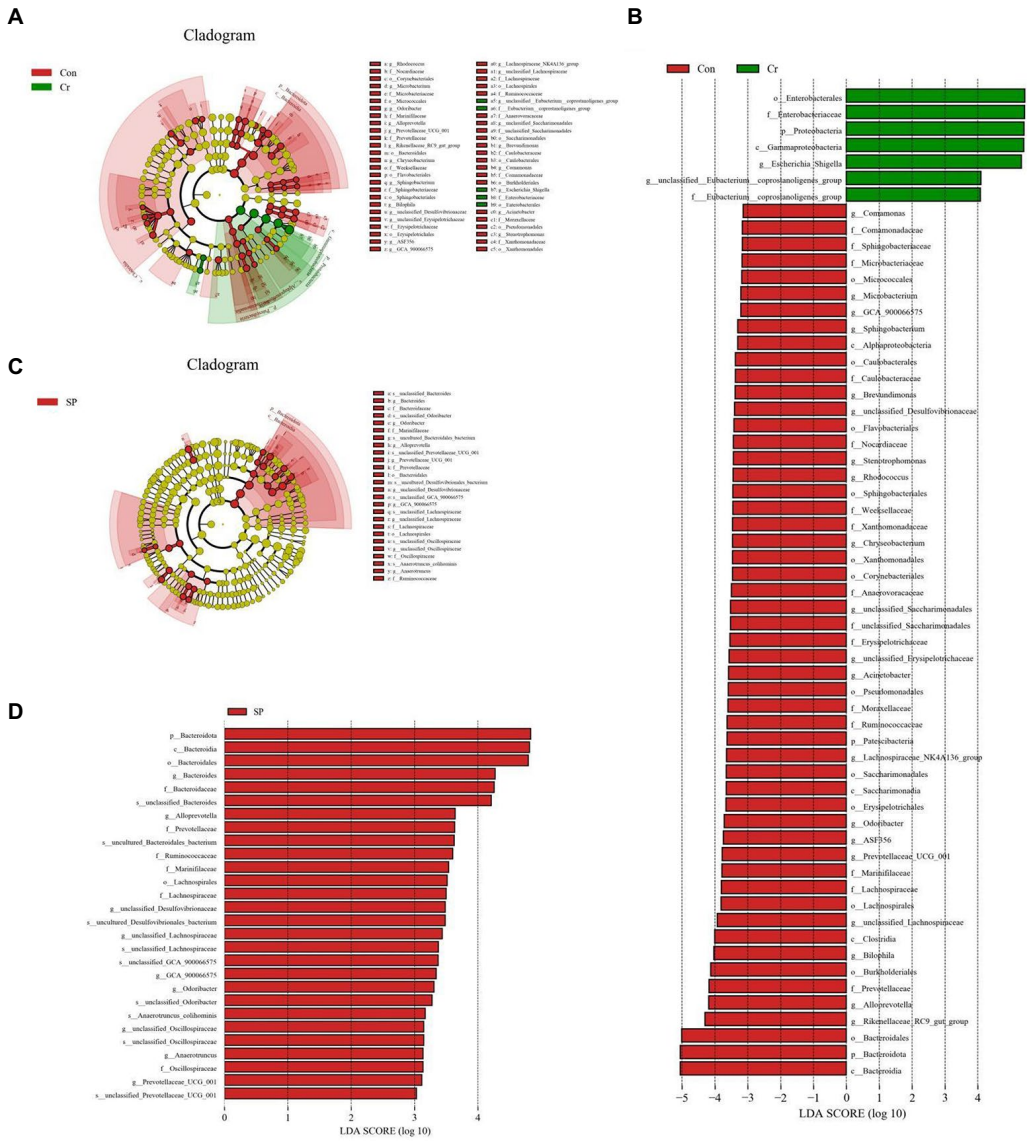
Identification of differential bacteria by LEfSe and LDA scores. **(A,C)** Cladogram of phylogenetic distribution of differential bacteria. **(B,D)** LDA scores >2 were considered significantly different.

At the phylum level, the abundances of *Proteobacteria* was observably more preponderant in Cr group than in the Con group, whereas the abundances of *Bacteroidota*, *Patescibacteria*, and *Actinobacteriota* were lower. At the genus level, the abundances of *Escherichia_Shigella* and *Enterococcus* in Cr group was observably predominant than Con group, whereas the *unclassified_Lachnospiraceae*, *Prevotellaceae_UCG_001*, *unclassified_Desulfovibrionaceae*, *Bilophila*, *Stenotrophomonas*, *Lachnoclostridium*, *Rikenellaceae_RC9_gut_group*, *Rhodococcus*, *Microbacterium*, *Candidatus_Saccharimonas*, *Enterorhabdus*, *unclassified_Erysipelotrichaceae*, *Sphingobacterium*, *unclassified_Anaerovoracaceae*, *Anaerotruncus*, *unclassified_Ruminococcaceae*, and *Acinetobacter* were lower (Figure 4). However, seaweed polysaccharide administration could reverse these bacterial changes. A comparison of the Cr and SP showed a distinct decrease in the abundances of *Bacteroides*, *Alloprevotella*, *unclassified_Desulfovibrionaceae*, *unclassified_Ruminococcaceae*, *Odoribacter*, *GCA_900066575*, *unclassified_Lachnospiraceae*, *Anaerotruncus*, and *Alistipes*. LEfSe analysis further revealed bacteria that differed between groups (Figure 5).

### Correlation network analysis

**Figure 6:**
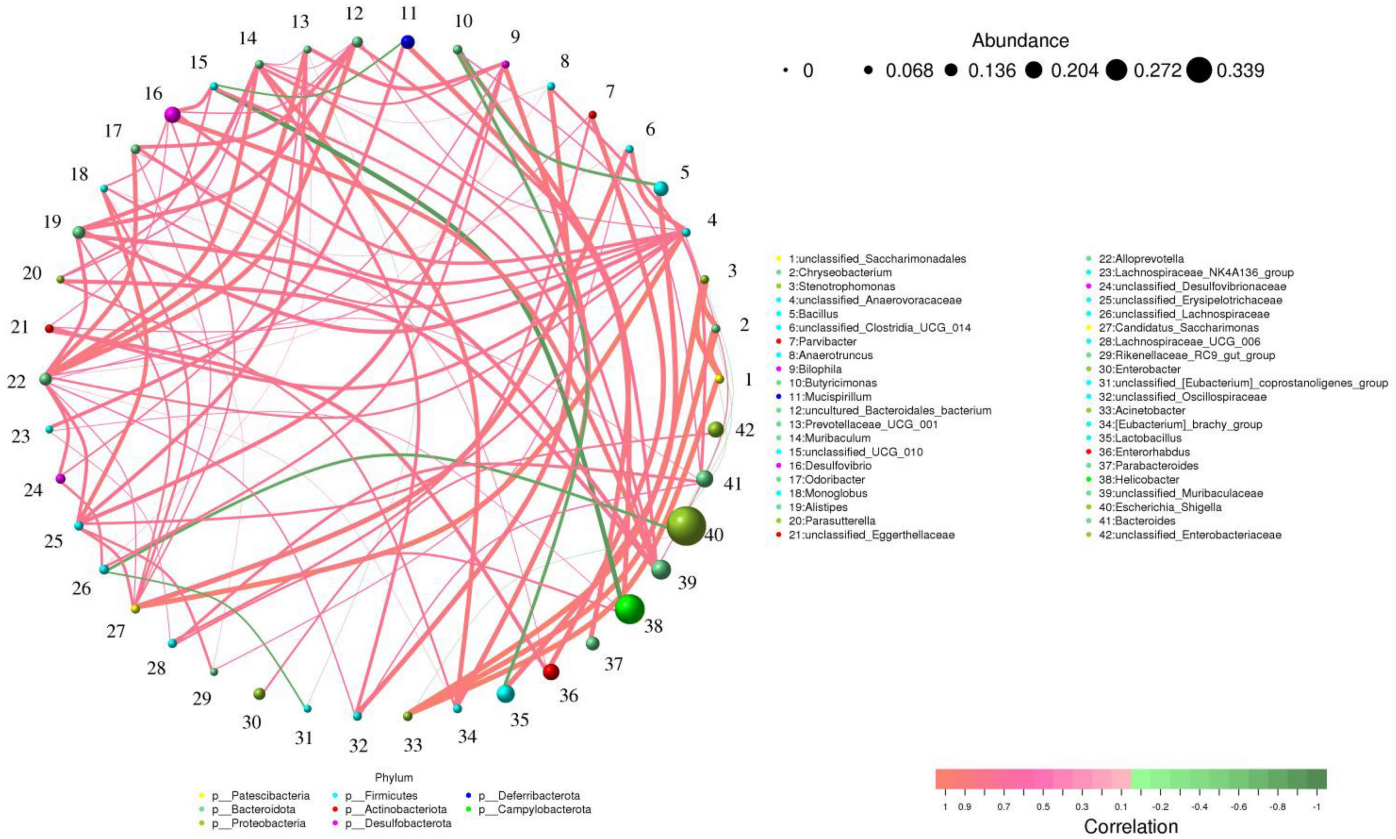
Correlation network analysis of gut microbiota. The green and red lines indicate the negative and positive correlation, respectively.

*Alistipes* was positively related to *unclassified_Muribaculaceae* (0.79). *Prevotellaceae_UCG_001* was positively related to *Alloprevotella* (0.83), *unclassified_Erysipelotrichaceae* (0.77) and *unclassified_Lachnospiraceae* (0.66). *Rikenellaceae_RC9_gut_group* was positively related to *unclassified_Erysipelotrichaceae* (0.72) and *unclassified_Clostridia_UCG_014* (0.67) (Figure 6).

## Discussion

The environmental contamination caused by heavy metal discharge and the negative impact on public health have attracted increasing attention (Drzezdzon et al., 2018; Yuan et al., 2020). In addition, the accumulation of heavy metals in animals and plants also seriously affects animal production and human health (Quina et al., 2019; Bao et al., 2021). Studies have shown that chromium could be absorbed by the host in many ways such as the digestive system, epidermis and respiratory system (Zhang et al., 2020). Moreover, chromium ingested by the digestive tract can enter other organs such as liver, kidney, and intestine through blood circulation, which further threatens the health of the host (Cardenas-Gonzalez et al., 2016; Andleeb et al., 2020). Early investigations showed that long-term chromium exposure can cause a significant decrease in growth performance and perturb gut microbial homeostasis in broilers (Li Y. et al., 2021). In addition, chromium exposure has also been shown to cause significant gastrointestinal symptoms (Zhang et al., 2022). It is widely known that the intestine plays an important role in nutrient absorption and host health, which in turn depends on normal gut microbiota structure (Brussow, 2015; Coelho et al., 2019). Although gut microbiota inhabits the intestine, it can cause systemic effects. Therefore, the maintenance of gut microbial homeostasis is critical for host health (Greenhill, 2018; Ma et al., 2020). Chromium intake through the digestive tract inevitably affects the gut microbiota and causes kidney damage, but whether seaweed polysaccharides with various biological properties can restore the gut microbiota is still unknown. Therefore, we systematically explored the protective effects of seaweed polysaccharides on hexavalent chromium-induced gut microbiota in mice.

As the main channel for various substances to enter the body, the intestinal own health and the gut microbiota inhabiting the intestine are also more susceptible to external factors (Sadeq et al., 2021; Zheng et al., 2021). Generally, the gut microbiota is in a dynamic balance under the action of various factors, but intestinal function does not change significantly (Michaudel and Sokol, 2020). In addition, the stability of the gut microbiota is also necessary to maintain the host health and the intestinal function (Li et al., 2016). However, environmental pollutants such as heavy metals, microplastics and pesticides can damage the intestine and various parenchymal organs, causing gut microbial imbalance and systemic effects (Lu et al., 2019; Qiao et al., 2019). Additionally, dysbiosis in the gut microbiota also affects the digestion and absorption of nutrients and growth performance (Chi et al., 2021). Previous study showed that long-term hexavalent chromium exposure leads to dysbiosis of the gut microbiota, accompanied by a significant reduction in microbial diversity (Li A. et al., 2022). Additionally, Yao et al. (2019) also found similar conclusions, demonstrating the negative impact of hexavalent chromium on gut microbes. In this study, we observed significant decrease in gut microbial diversity of mice during hexavalent chromium exposure. However, seaweed polysaccharide administration could restore the gut microbial dysbiosis caused by chromium exposure. Studies have shown that gut microbial dysbiosis and reduced diversity are considered important drivers of various diseases such as diarrhea, obesity and diabetes (Stephens et al., 2018; Lee P. et al., 2019). Moreover, decreased gut microbial diversity also affects intestinal barrier function and immune system maturation, which may reduce host immunity and increase permeability (Burcelin, 2016; Van Averbeke et al., 2022). In this case, the host is more sensitive to external pathogenic factors and more prone to other diseases. Increased intestinal permeability may also cause the passage of harmful intestinal metabolites or pathogenic bacteria across the intestinal barrier, leading to damage to other organs such as liver and kidney (Adolph and Tilg, 2018; Wahlstrom, 2019). More importantly, some opportunistic pathogens may also become pathogenic during this period (Nishida et al., 2018). Therefore, maintaining the balance of gut microbiota is also considered to be an important condition to ensure the health of the host. In addition, we also performed beta diversity analysis to explore the differences in the main components of the gut microbiota. Results showed that all the samples were clustered together, indicating that there were no differences in the main components of the gut microbiota.

As the most complex micro-ecosystem, the gut microbiota is composed of a large number of microorganisms, of which bacteria account for approximately 98% (Qin et al., 2022; Yakabe et al., 2022). Intestinal bacteria play key roles in intestinal function and homeostasis by interacting with the host or producing some beneficial metabolites (Haase et al., 2020; Zhou et al., 2022). Consistent with previous studies, we observed that hexavalent chromium exposure could cause significant changes in gut microbial composition, indicating the disruption of gut microbial homeostasis. Specifically, hexavalent chromium exposure led to a significant increase in gut pathogenic bacteria (*Enterococcus* and *Escherichia_Shigella*) and a significant decrease in beneficial bacteria (*Alistipes*, *Lachnospiraceae*, *Prevotellaceae_UCG_001*, *Alloprevotella*, *Bacteroides* and *Rikenellaceae_RC9_gut_group*). However, seaweed polysaccharide administration significantly improved the composition of the gut microbiota in mice. Studies have shown that *Alistipes* and *Lachnospiraceae* could produce short-chain fatty acids (SCFAs; Wu et al., 2020). Many investigations indicate that SCFAs played vital roles in relieving intestinal inflammation, oxidative stress, opportunistic infections as well as maintaining gut microbial homeostasis, intestinal permeability and intestinal epithelial cells morphology (Marino et al., 2017; Schwarz et al., 2017; Ikeda et al., 2022). Moreover, SCFAs has also been shown to regulate energy intake, regulate cell apoptosis and decrease cholesterol (Murugesan et al., 2018; Prasad and Bondy, 2018; Yang J. et al., 2022; Yang X. et al., 2022). *Prevotellaceae* in the intestine could digest pectin, hemicellulose and high carbohydrate foods, indicating its key roles in digestion and absorption (Li A. et al., 2022; Li C. et al., 2022). *Alloprevotella* could secrete acetate and succinate and these beneficial metabolites are critical for intestinal homeostasis and decreased cardiovascular disease risk (Yuan et al., 2021). *Bacteroides* could decompose polysaccharides, showing a key role in intestinal ecosystem (Schwalm et al., 2016; Schwalm and Groisman, 2017). *Rikenellaceae* could alleviate inflammation by activating T-regulatory cell differentiation (Cui et al., 2018). Numerous evidence demonstrate that *Enterococcus* could cause many diseases such as meningitis, sepsis, and cardioperiostitis (Su et al., 2016; Subramanya et al., 2019). Additionally, *Enterococcus* infection is difficult to cure because of inherent and acquired resistance (Chanderraj et al., 2020; Ekore et al., 2022). *Escherichia_Shigella* was considered as a vital factor for causing diarrhea (Li et al., 2018). Hexavalent chromium exposure may further adversely affect host health by disrupting gut microbial homeostasis. However, seaweed polysaccharide can maintain the gut microbial balance and this may be one of the modes of action of seaweed polysaccharides. Microorganisms inhabiting the intestine could interact in a synergistic, antagonistic or symbiotic relationship to form a stable intestinal environment (Li A. et al., 2022; Li C. et al., 2022). In this study, we observed significant correlations among some bacteria through correlation network analysis. For instance, *Alistipes* and *Prevotellaceae_UCG_001* were associated with *unclassified_Muribaculaceae* and *Alloprevotella*, respectively. Therefore, these altered bacteria may further affect the function of other bacteria through the interaction between bacteria.

## Conclusion

In conclusion, this research explored the protective effect of seaweed polysaccharide administration on the gut microbiota of hexavalent chromium-exposed mice. Results showed that seaweed polysaccharide administration could alleviate hexavalent chromium exposure induced gut microbiota dysbiosis. Our study shows that seaweed polysaccharides can be used as an effective drug to mitigate the negative effects of hexavalent chromium exposure on host health. Meanwhile, maintaining the homeostasis of gut microbiota may be one of the ways that seaweed polysaccharides exert their pharmacological effects.

## Data availability statement

The original sequence data was submitted to the Sequence Read Archive (SRA) (NCBI, USA) with the accession no. PRJNA902534.

## Ethics statement

The animal study was reviewed and approved by the study was conducted under the guidance and approval of the Animal Welfare and Ethics Committee of Henan university of Animal Husbandry and Economy.

## Author contributions

JM, ZG, and XM provided the idea. XiuW, XueW, YF, and XL contributed reagents, materials, and analysis tools. JM wrote the manuscript. XueW, YF, XL, FZ, GH, and XM revised the manuscript. All authors contributed to the article and approved the submitted version.

## Funding

This work was supported by Key Discipline of Veterinary Medicine of Henan University of Animal Husbandry and Economy (XJXK202202).

## Conflict of interest

The authors declare that the research was conducted in the absence of any commercial or financial relationships that could be construed as a potential conflict of interest.

## Publisher’s note

All claims expressed in this article are solely those of the authors and do not necessarily represent those of their affiliated organizations, or those of the publisher, the editors and the reviewers. Any product that may be evaluated in this article, or claim that may be made by its manufacturer, is not guaranteed or endorsed by the publisher.

## References

[ref1] Adolph T. E. Tilg H. (2018). Gut microbiota as gatekeeper of anti-tumour responses in the liver. Nat. Rev. Gastroenterol. Hepatol. 15, 584–586. doi: 10.1038/s41575-018-0046-1, PMID: 30013103

[ref2] Andleeb S. Ahmad Z. Mahmood T. Bao S. Arif S. A. Jha S. K. (2020). Evaluating toxicity impacts of environmental exposed chromium on small Indian mongoose (*Urva auropunctatus*) hematological, biochemical and histopathological functioning. Chemosphere 259:127485. doi: 10.1016/j.chemosphere.2020.127485, PMID: 32650164

[ref3] Bao X. Asgari A. Najafi M. L. Mokammel A. Ahmadi M. Akbari S.. (2021). Exposure to waterpipe smoke and blood heavy metal concentrations. Environ. Res. 200:111460. doi: 10.1016/j.envres.2021.111460, PMID: 34089744

[ref4] Bauer S. Jin W. Zhang F. Linhardt R. J. (2021). The application of seaweed polysaccharides and their derived products with potential for the treatment of Alzheimer's disease. Mar. Drugs 19:2021. doi: 10.3390/md19020089, PMID: 33557077 PMC7913876

[ref5] Ben H. F. Troudi A. Sefi M. Boudawara T. Zeghal N. (2016). The protective effect of propylthiouracil against hepatotoxicity induced by chromium in adult mice. Toxicol. Ind. Health 32, 235–245. doi: 10.1177/0748233713498446, PMID: 24081637

[ref6] Brussow H. (2015). Growth promotion and gut microbiota: insights from antibiotic use. Environ. Microbiol. 17, 2216–2227. doi: 10.1111/1462-2920.12786, PMID: 25627910

[ref7] Burcelin R. (2016). Gut microbiota and immune crosstalk in metabolic disease. Mol. Metab. 5, 771–781. doi: 10.1016/j.molmet.2016.05.016, PMID: 27617200 PMC5004167

[ref8] Cardenas-Gonzalez M. Osorio-Yanez C. Gaspar-Ramirez O. Pavkovic M. Ochoa-Martinez A. Lopez-Ventura D.. (2016). Environmental exposure to arsenic and chromium in children is associated with kidney injury molecule-1. Environ. Res. 150, 653–662. doi: 10.1016/j.envres.2016.06.032, PMID: 27431456 PMC5003729

[ref9] Chanderraj R. Brown C. A. Hinkle K. Falkowski N. Ranjan P. Dickson R. P.. (2020). Gut microbiota predict enterococcus expansion but not vancomycin-resistant enterococcus acquisition. mSphere 5, 665–669. doi: 10.1128/mSphere.00537-20, PMID: 33208515 PMC7677005

[ref10] Chen F. Huang G. (2018). Preparation and immunological activity of polysaccharides and their derivatives. Int. J. Biol. Macromol. 112, 211–216. doi: 10.1016/j.ijbiomac.2018.01.169 29382579

[ref11] Chen F. Huang G. (2019). Antioxidant activity of polysaccharides from different sources of ginseng. Int. J. Biol. Macromol. 125, 906–908. doi: 10.1016/j.ijbiomac.2018.12.134, PMID: 30572039

[ref12] Chi L. Tu P. Ru H. Lu K. (2021). Studies of xenobiotic-induced gut microbiota dysbiosis: from correlation to mechanisms. Gut Microbes 13:1921912. doi: 10.1080/19490976.2021.1921912, PMID: 34313531 PMC8346244

[ref13] Coelho O. Candido F. G. Alfenas R. (2019). Dietary fat and gut microbiota: mechanisms involved in obesity control. Crit. Rev. Food Sci. Nutr. 59, 3045–3053. doi: 10.1080/10408398.2018.1481821, PMID: 29851507

[ref14] Crusell M. Hansen T. H. Nielsen T. Allin K. H. Ruhlemann M. C. Damm P.. (2018). Gestational diabetes is associated with change in the gut microbiota composition in third trimester of pregnancy and postpartum. Microbiome 6:89. doi: 10.1186/s40168-018-0472-x, PMID: 29764499 PMC5952429

[ref15] Cui H. X. Hu Y. N. Li J. W. Yuan K. (2018). Hypoglycemic mechanism of the Berberine organic acid salt under the synergistic effect of intestinal Flora and oxidative stress. Oxidative Med. Cell. Longev. 2018, 8930374–8930313. doi: 10.1155/2018/8930374, PMID: 30662584 PMC6313974

[ref16] Drzezdzon J. Jacewicz D. Chmurzynski L. (2018). The impact of environmental contamination on the generation of reactive oxygen and nitrogen species – consequences for plants and humans. Environ. Int. 119, 133–151. doi: 10.1016/j.envint.2018.06.019, PMID: 29957355

[ref17] Egerton S. Culloty S. Whooley J. Stanton C. Ross R. P. (2018). The gut microbiota of marine fish. Front. Microbiol. 9:873. doi: 10.3389/fmicb.2018.00873, PMID: 29780377 PMC5946678

[ref18] Ekore D. O. Onanga R. Nguema P. Lozano C. Kumulungui B. S. (2022). The antibiotics used in livestock and their impact on resistance in *Enterococcus faecium* and *Enterococcus hirae* on farms in Gabon. Antibiotics 11:224. doi: 10.3390/antibiotics11020224, PMID: 35203826 PMC8868485

[ref19] Feng Q. Chen W. D. Wang Y. D. (2018). Gut microbiota: an integral moderator in health and disease. Front. Microbiol. 9:151. doi: 10.3389/fmicb.2018.00151, PMID: 29515527 PMC5826318

[ref20] Fu X. Zhan Y. Li N. Yu D. Gao W. Gu Z.. (2021). Enzymatic preparation of low-molecular-weight *Laminaria japonica* polysaccharides and evaluation of its effect on modulating intestinal microbiota in high-fat-diet-fed mice. Front. Bioeng. Biotechnol. 9:820892. doi: 10.3389/fbioe.2021.820892, PMID: 35237590 PMC8883051

[ref21] Greenhill C. (2018). Exercise affects gut microbiota and bone. Nat. Rev. Endocrinol. 14:322. doi: 10.1038/s41574-018-0014-4, PMID: 29692411

[ref22] Haase S. Wilck N. Haghikia A. Gold R. Mueller D. N. Linker R. A. (2020). The role of the gut microbiota and microbial metabolites in neuroinflammation. Eur. J. Immunol. 50, 1863–1870. doi: 10.1002/eji.201847807 33188704

[ref23] He Y. Zou L. Luo W. Yi Z. Yang P. Yu S.. (2020). Heavy metal exposure, oxidative stress and semen quality: exploring associations and mediation effects in reproductive-aged men. Chemosphere 244:125498. doi: 10.1016/j.chemosphere.2019.125498, PMID: 31812049

[ref24] Ikeda T. Nishida A. Yamano M. Kimura I. (2022). Short-chain fatty acid receptors and gut microbiota as therapeutic targets in metabolic, immune, and neurological diseases. Pharmacol. Ther. 239:108273. doi: 10.1016/j.pharmthera.2022.108273, PMID: 36057320

[ref25] Kakade A. Salama E. S. Pengya F. Liu P. Li X. (2020). Long-term exposure of high concentration heavy metals induced toxicity, fatality, and gut microbial dysbiosis in common carp, *Cyprinus carpio*. Environ. Pollut. 266:115293. doi: 10.1016/j.envpol.2020.115293, PMID: 32781213

[ref26] Kapoor R. T. Bani M. M. Alam P. Rinklebe J. Ahmad P. (2022). Accumulation of chromium in plants and its repercussion in animals and humans. Environ. Pollut. 301:119044. doi: 10.1016/j.envpol.2022.119044, PMID: 35217142

[ref27] Lee C. P. Hsu P. Y. Su C. C. (2019). Increased prevalence of Sjogren's syndrome in where soils contain high levels of chromium. Sci. Total Environ. 657, 1121–1126. doi: 10.1016/j.scitotenv.2018.12.122, PMID: 30677879

[ref28] Lee P. Yacyshyn B. R. Yacyshyn M. B. (2019). Gut microbiota and obesity: an opportunity to alter obesity through faecal microbiota transplant (FMT). Diabetes Obes. Metab. 21, 479–490. doi: 10.1111/dom.13561, PMID: 30328245

[ref29] Li C. Chen N. Zhang X. Shahzad K. Qi R. Zhang Z.. (2022). Mixed silage with Chinese cabbage waste enhances antioxidant ability by increasing ascorbate and aldarate metabolism through rumen Prevotellaceae UCG-004 in Hu sheep. Front. Microbiol. 13:978940. doi: 10.3389/fmicb.2022.978940, PMID: 36090065 PMC9459383

[ref30] Li A. Ding J. Shen T. Han Z. Zhang J. Abadeen Z. U.. (2021). Environmental hexavalent chromium exposure induces gut microbial dysbiosis in chickens. Ecotoxicol. Environ. Saf. 227:112871. doi: 10.1016/j.ecoenv.2021.112871, PMID: 34649138

[ref31] Li Y. Hu X. Yang S. Zhou J. Qi L. Sun X.. (2018). Comparison between the fecal bacterial microbiota of healthy and diarrheic captive musk deer. Front. Microbiol. 9:300. doi: 10.3389/fmicb.2018.00300, PMID: 29551996 PMC5840195

[ref32] Li A. Wang Y. Hao J. Wang L. Quan L. Duan K.. (2022). Long-term hexavalent chromium exposure disturbs the gut microbial homeostasis of chickens. Ecotoxicol. Environ. Saf. 237:113532. doi: 10.1016/j.ecoenv.2022.113532, PMID: 35472558

[ref33] Li D. Wang P. Wang P. Hu X. Chen F. (2016). The gut microbiota: a treasure for human health. Biotechnol. Adv. 34, 1210–1224. doi: 10.1016/j.biotechadv.2016.08.003, PMID: 27592384

[ref34] Li Y. Xia S. Jiang X. Feng C. Gong S. Ma J.. (2021). Gut microbiota and diarrhea: an updated review. Front. Cell. Infect. Microbiol. 11:625210. doi: 10.3389/fcimb.2021.625210, PMID: 33937093 PMC8082445

[ref35] Liu C. S. Liang X. Wei X. H. Jin Z. Chen F. L. Tang Q. F.. (2019). Gegen Qinlian decoction treats diarrhea in piglets by modulating gut microbiota and short-chain fatty acids. Front. Microbiol. 10:825. doi: 10.3389/fmicb.2019.00825, PMID: 31057525 PMC6482297

[ref36] Lomartire S. Goncalves A. (2022). Novel technologies for seaweed polysaccharides extraction and their use in food with therapeutically applications-a review. Foods 11:2654. doi: 10.3390/foods11172654, PMID: 36076839 PMC9455623

[ref37] Lu L. Luo T. Zhao Y. Cai C. Fu Z. Jin Y. (2019). Interaction between microplastics and microorganism as well as gut microbiota: a consideration on environmental animal and human health. Sci. Total Environ. 667, 94–100. doi: 10.1016/j.scitotenv.2019.02.380, PMID: 30826685

[ref38] Ma Z. Gao X. Yang X. Lin L. Wei X. Wang S.. (2022). Low-dose florfenicol and copper combined exposure during early life induced health risks by affecting gut microbiota and metabolome in SD rats. Ecotoxicol. Environ. Saf. 245:114120. doi: 10.1016/j.ecoenv.2022.114120, PMID: 36174320

[ref39] Ma T. Villot C. Renaud D. Skidmore A. Chevaux E. Steele M.. (2020). Linking perturbations to temporal changes in diversity, stability, and compositions of neonatal calf gut microbiota: prediction of diarrhea. ISME J. 14, 2223–2235. doi: 10.1038/s41396-020-0678-3, PMID: 32444812 PMC7609338

[ref40] Mamais D. Noutsopoulos C. Kavallari I. Nyktari E. Kaldis A. Panousi E.. (2016). Biological groundwater treatment for chromium removal at low hexavalent chromium concentrations. Chemosphere 152, 238–244. doi: 10.1016/j.chemosphere.2016.02.124, PMID: 26971177

[ref41] Marino E. Richards J. L. Mcleod K. H. Stanley D. Yap Y. A. Knight J.. (2017). Gut microbial metabolites limit the frequency of autoimmune T cells and protect against type 1 diabetes. Nat. Immunol. 18, 552–562. doi: 10.1038/ni.3713, PMID: 28346408

[ref42] Michaudel C. Sokol H. (2020). The gut microbiota at the service of immunometabolism. Cell Metab. 32, 514–523. doi: 10.1016/j.cmet.2020.09.004, PMID: 32946809

[ref43] Monteiro J. Cunha L. Costa M. Reis H. Aguiar A. Oliveira-Bahia V.. (2018). Mutagenic and histopathological effects of hexavalent chromium in tadpoles of Lithobates catesbeianus (Shaw, 1802) (Anura, Ranidae). Ecotoxicol. Environ. Saf. 163, 400–407. doi: 10.1016/j.ecoenv.2018.07.083, PMID: 30064085

[ref44] Morris A. (2018). Gut microbiota: fibre restores healthy gut microbiota. Nat. Rev. Endocrinol. 14:63. doi: 10.1038/nrendo.2017.182 29286051

[ref45] Murugesan S. Nirmalkar K. Hoyo-Vadillo C. Garcia-Espitia M. Ramirez-Sanchez D. Garcia-Mena J. (2018). Gut microbiome production of short-chain fatty acids and obesity in children. Eur. J. Clin. Microbiol. Infect. Dis. 37, 621–625. doi: 10.1007/s10096-017-3143-0, PMID: 29196878

[ref46] Nguyen K. L. Nguyen H. A. Richter O. Pham M. T. Nguyen V. P. (2017). Ecophysiological responses of young mangrove species *Rhizophora apiculata* (Blume) to different chromium contaminated environments. Sci. Total Environ. 574, 369–380. doi: 10.1016/j.scitotenv.2016.09.063, PMID: 27639473

[ref47] Nishida A. Inoue R. Inatomi O. Bamba S. Naito Y. Andoh A. (2018). Gut microbiota in the pathogenesis of inflammatory bowel disease. Clin. J. Gastroenterol. 11, 1–10. doi: 10.1007/s12328-017-0813-5 29285689

[ref48] Paithankar J. G. Saini S. Dwivedi S. Sharma A. Chowdhuri D. K. (2021). Heavy metal associated health hazards: An interplay of oxidative stress and signal transduction. Chemosphere 262:128350. doi: 10.1016/j.chemosphere.2020.128350, PMID: 33182141

[ref49] Prasad K. N. Bondy S. C. (2018). Dietary fibers and their fermented short-chain fatty acids in prevention of human diseases. Mech. Ageing Dev. 17:100170. doi: 10.1016/j.mad.2018.10.003, PMID: 30336163

[ref50] Prasad S. Yadav K. K. Kumar S. Gupta N. Cabral-Pinto M. Rezania S.. (2021). Chromium contamination and effect on environmental health and its remediation: a sustainable approaches. J. Environ. Manag. 285:112174. doi: 10.1016/j.jenvman.2021.112174, PMID: 33607566

[ref51] Qiao R. Deng Y. Zhang S. Wolosker M. B. Zhu Q. Ren H.. (2019). Accumulation of different shapes of microplastics initiates intestinal injury and gut microbiota dysbiosis in the gut of zebrafish. Chemosphere 236:124334. doi: 10.1016/j.chemosphere.2019.07.065, PMID: 31310986

[ref52] Qin D. Bai Y. Li Y. Huang Y. Li L. Wang G.. (2022). Changes in gut microbiota by the lactobacillus casei anchoring the K88 Fimbrial protein prevented newborn piglets from clinical diarrhea. Front. Cell. Infect. Microbiol. 12:842007. doi: 10.3389/fcimb.2022.842007, PMID: 35372106 PMC8972131

[ref53] Quina A. S. Durao A. F. Munoz-Munoz F. Ventura J. Da L. M. M. (2019). Population effects of heavy metal pollution in wild Algerian mice (*Mus spretus*). Ecotoxicol. Environ. Saf. 171, 414–424. doi: 10.1016/j.ecoenv.2018.12.062, PMID: 30639867

[ref54] Sadeq S. A. Mills R. Beckerman A. P. (2021). The microbiome mediates the interaction between predation and heavy metals. Sci. Total Environ. 775:145144. doi: 10.1016/j.scitotenv.2021.145144, PMID: 33631565

[ref55] Schwalm N. R. Groisman E. A. (2017). Navigating the gut buffet: control of polysaccharide utilization in *Bacteroides* spp. Trends Microbiol. 25, 1005–1015. doi: 10.1016/j.tim.2017.06.009, PMID: 28733133

[ref56] Schwalm N. R. Townsend G. N. Groisman E. A. (2016). Multiple signals govern utilization of a polysaccharide in the gut bacterium Bacteroides thetaiotaomicron. MBio 7, 1–16. doi: 10.1128/mBio.01342-16, PMID: 27729509 PMC5061871

[ref57] Schwarz A. Bruhs A. Schwarz T. (2017). The short-chain fatty acid sodium butyrate functions as a regulator of the skin immune system. J. Invest. Dermatol. 137, 855–864. doi: 10.1016/j.jid.2016.11.014, PMID: 27887954

[ref58] Shaw P. Mondal P. Bandyopadhyay A. Chattopadhyay A. (2019). Environmentally relevant concentration of chromium activates Nrf 2 and alters transcription of related XME genes in liver of zebrafish. Chemosphere 214, 35–46. doi: 10.1016/j.chemosphere.2018.09.104, PMID: 30253254

[ref59] Stephens R. W. Arhire L. Covasa M. (2018). Gut microbiota: from microorganisms to metabolic organ influencing obesity. Obesity 26, 801–809. doi: 10.1002/oby.22179, PMID: 29687647

[ref60] Su P. Y. Miller S. Rutishauser R. L. Babik J. (2016). Broad-range PCR for early diagnosis of nosocomial enterococcus gallinarum meningitis. Infect. Dis. Ther. 48, 640–642. doi: 10.3109/23744235.2016.1160422 27097275

[ref61] Subramanya S. H. Amberpet R. Chaudhary D. Nayak N. Padukone S. Bairy I.. (2019). Neonatal sepsis due to glycopeptide resistant *Enterococcus faecium* from colonized maternal gut-rare case evidence. Antimicrob. Resist. Infect. Control 8:29. doi: 10.1186/s13756-019-0490-x, PMID: 30774945 PMC6368750

[ref62] Sun X. Chen J. Huang Y. Zhu S. Wang S. Xu Z.. (2022). Yishen Qingli Heluo granule ameliorates renal dysfunction in 5/6 Nephrectomized rats by targeting gut microbiota and intestinal barrier integrity. Front. Pharmacol. 13:858881. doi: 10.3389/fphar.2022.858881, PMID: 35814258 PMC9258868

[ref63] Tanna B. Mishra A. (2019). Nutraceutical potential of seaweed polysaccharides: structure, bioactivity, safety, and toxicity. Compr. Rev. Food Sci. Food Saf. 18, 817–831. doi: 10.1111/1541-4337.12441, PMID: 33336929

[ref64] Vaiopoulou E. Gikas P. (2020). Regulations for chromium emissions to the aquatic environment in Europe and elsewhere. Chemosphere 254:126876. doi: 10.1016/j.chemosphere.2020.126876, PMID: 32957286

[ref65] Van Averbeke V. Berkell M. Mysara M. Rodriguez-Ruiz J. P. Xavier B. B. De Winter F.. (2022). Host immunity influences the composition of murine gut microbiota. Front. Immunol. 13:828016. doi: 10.3389/fimmu.2022.828016, PMID: 35371073 PMC8965567

[ref66] Wahlstrom A. (2019). Outside the liver box: the gut microbiota as pivotal modulator of liver diseases. Biochim. Biophys. Acta Mol. basis Dis. 1865, 912–919. doi: 10.1016/j.bbadis.2018.07.004, PMID: 31007175

[ref67] Wan J. Ma J. (2022). Efficacy of dietary supplements targeting gut microbiota in the prevention and treatment of gestational diabetes mellitus. Front. Microbiol. 13:927883. doi: 10.3389/fmicb.2022.927883, PMID: 35910625 PMC9330481

[ref68] Wang F. Huo L. Li Y. Wu L. Zhang Y. Shi G.. (2022). A hybrid framework for delineating the migration route of soil heavy metal pollution by heavy metal similarity calculation and machine learning method. Sci. Total Environ. 858:160065. doi: 10.1016/j.scitotenv.2022.160065, PMID: 36356739

[ref69] Wang R. Yang X. Liu J. Zhong F. Zhang C. Chen Y.. (2022). Gut microbiota regulates acute myeloid leukaemia via alteration of intestinal barrier function mediated by butyrate. Nat. Commun. 13:2522. doi: 10.1038/s41467-022-30240-8, PMID: 35534496 PMC9085760

[ref70] Wen X. Lu J. Wu J. Lin Y. Luo Y. (2019). Influence of coastal groundwater salinization on the distribution and risks of heavy metals. Sci. Total Environ. 652, 267–277. doi: 10.1016/j.scitotenv.2018.10.250, PMID: 30366327

[ref71] Wu M. Yang S. Wang S. Cao Y. Zhao R. Li X.. (2020). Effect of Berberine on atherosclerosis and gut microbiota modulation and their correlation in high-fat diet-fed ApoE−/− mice. Front. Pharmacol. 11:223. doi: 10.3389/fphar.2020.00223, PMID: 32231564 PMC7083141

[ref72] Xia J. Jin C. Pan Z. Sun L. Fu Z. Jin Y. (2018). Chronic exposure to low concentrations of lead induces metabolic disorder and dysbiosis of the gut microbiota in mice. Sci. Total Environ. 631–632, 439–448. doi: 10.1016/j.scitotenv.2018.03.053, PMID: 29529432

[ref73] Xu Y. Jing H. Wang J. Zhang S. Chang Q. Li Z.. (2022). Disordered gut microbiota correlates with altered fecal bile acid metabolism and post-cholecystectomy diarrhea. Front. Microbiol. 13:800604. doi: 10.3389/fmicb.2022.800604, PMID: 35250923 PMC8894761

[ref74] Yakabe K. Higashi S. Akiyama M. Mori H. Murakami T. Toyoda A.. (2022). Dietary-protein sources modulate host susceptibility to *Clostridioides difficile* infection through the gut microbiota. Cell Rep. 40:111332. doi: 10.1016/j.celrep.2022.111332, PMID: 36103838

[ref75] Yang X. Ai P. He X. Mo C. Zhang Y. Xu S.. (2022). Parkinson's disease is associated with impaired gut-blood barrier for short-chain fatty acids. Mov. Disord. 37, 1634–1643. doi: 10.1002/mds.29063 35607987

[ref76] Yang Q. Han B. Xue J. Lv Y. Li S. Liu Y.. (2020). Hexavalent chromium induces mitochondrial dynamics disorder in rat liver by inhibiting AMPK/PGC-1alpha signaling pathway. Environ. Pollut. 265:114855. doi: 10.1016/j.envpol.2020.114855, PMID: 32474337

[ref77] Yang G. Wei J. Liu P. Zhang Q. Tian Y. Hou G.. (2021). Role of the gut microbiota in type 2 diabetes and related diseases. Metabolism 117:154712. doi: 10.1016/j.metabol.2021.154712 33497712

[ref78] Yang J. Wei H. Zhou Y. Szeto C. H. Li C. Lin Y.. (2022). High-fat diet promotes colorectal tumorigenesis through modulating gut microbiota and metabolites. Gastroenterology 162, 135.e2–149.e2. doi: 10.1053/j.gastro.2021.08.041, PMID: 34461052

[ref79] Yang Y. Zhang W. Wang S. Zhang H. Zhang Y. (2020). Response of male reproductive function to environmental heavy metal pollution in a free-living passerine bird, *Passer montanus*. Sci. Total Environ. 747:141402. doi: 10.1016/j.scitotenv.2020.141402, PMID: 32771794

[ref80] Yao Q. Yang H. Wang X. Wang H. (2019). Effects of hexavalent chromium on intestinal histology and microbiota in Bufo gargarizans tadpoles. Chemosphere 216, 313–323. doi: 10.1016/j.chemosphere.2018.10.147, PMID: 30384300

[ref81] Ye J. Wu Z. Zhao Y. Zhang S. Liu W. Su Y. (2022). Role of gut microbiota in the pathogenesis and treatment of diabetes mullites: advanced research-based review. Front. Microbiol. 13:1029890. doi: 10.3389/fmicb.2022.1029890, PMID: 36338058 PMC9627042

[ref82] Yu X. Wu Z. Song Z. Zhang H. Zhan J. Yu H.. (2020). Single-anastomosis duodenal Jejunal bypass improve glucose metabolism by regulating gut microbiota and short-chain fatty acids in Goto-Kakisaki rats. Front. Microbiol. 11:273. doi: 10.3389/fmicb.2020.00273, PMID: 32153548 PMC7047167

[ref83] Yuan Y. Lu L. Bo N. Chaoyue Y. Haiyang Y. (2021). Allicin ameliorates intestinal barrier damage via microbiota-regulated short-chain fatty acids-TLR4/MyD88/NF-kappaB Cascade response in acrylamide-induced rats. J. Agric. Food Chem. 69, 12837–12852. doi: 10.1021/acs.jafc.1c05014, PMID: 34694121

[ref84] Yuan W. Zhou Y. Chen Y. Liu X. Wang J. (2020). Toxicological effects of microplastics and heavy metals on the Daphnia magna. Sci. Total Environ. 746:141254. doi: 10.1016/j.scitotenv.2020.141254, PMID: 32768788

[ref85] Zhang Z. Cao H. Song N. Zhang L. Cao Y. Tai J. (2020). Long-term hexavalent chromium exposure facilitates colorectal cancer in mice associated with changes in gut microbiota composition. Food Chem. Toxicol. 138:111237. doi: 10.1016/j.fct.2020.111237, PMID: 32145354

[ref86] Zhang X. Coker O. O. Chu E. S. Fu K. Lau H. Wang Y. X.. (2021). Dietary cholesterol drives fatty liver-associated liver cancer by modulating gut microbiota and metabolites. Gut 70, 761–774. doi: 10.1136/gutjnl-2019-319664, PMID: 32694178 PMC7948195

[ref87] Zhang L. Jing J. Han L. Wang J. Zhang W. Liu Z.. (2021). Characterization of gut microbiota, metabolism and cytokines in benzene-induced hematopoietic damage. Ecotoxicol. Environ. Saf. 228:112956. doi: 10.1016/j.ecoenv.2021.112956, PMID: 34781132

[ref88] Zhang Y. Long C. Hu G. Hong S. Su Z. Zhang Q.. (2022). Two-week repair alleviates hexavalent chromium-induced hepatotoxicity, hepatic metabolic and gut microbial changes: a dynamic inhalation exposure model in male mice. Sci. Total Environ. 857:159429. doi: 10.1016/j.scitotenv.2022.159429, PMID: 36243064

[ref89] Zhao X. Joo J. C. Lee J. K. Kim J. Y. (2019). Mathematical estimation of heavy metal accumulations in Helianthus annuus L. with a sigmoid heavy metal uptake model. Chemosphere 220, 965–973. doi: 10.1016/j.chemosphere.2018.12.210, PMID: 33395818

[ref90] Zheng R. Wang P. Cao B. Wu M. Li X. Wang H.. (2021). Intestinal response characteristic and potential microbial dysbiosis in digestive tract of Bufo gargarizans after exposure to cadmium and lead, alone or combined. Chemosphere 271:129511. doi: 10.1016/j.chemosphere.2020.129511 33445016

[ref91] Zhou J. Wu X. Li Z. Zou Z. Dou S. Li G.. (2022). Alterations in gut microbiota are correlated with serum metabolites in patients with insomnia disorder. Front. Cell. Infect. Microbiol. 12:722662. doi: 10.3389/fcimb.2022.722662, PMID: 35252021 PMC8892143

